# What Are the Factors Influencing Implementation of Advanced Access in Family Medicine Units? A Cross-Case Comparison of Four Early Adopters in Quebec

**DOI:** 10.1155/2017/1595406

**Published:** 2017-07-10

**Authors:** Sabina Abou Malham, Nassera Touati, Lara Maillet, Isabelle Gaboury, Christine Loignon, Mylaine Breton

**Affiliations:** ^1^Charles LeMoyne Hospital Research Center, Longueuil, QC, Canada; ^2^Université de Sherbrooke, Sherbrooke, QC, Canada; ^3^École Nationale d'Administration Publique, Montréal, QC, Canada; ^4^Institut Universitaire de Première Ligne en Santé et Services Sociaux, Sherbrooke, QC, Canada

## Abstract

**Introduction:**

Advanced access is an organizational model that has shown promise in improving timely access to primary care. In Quebec, it has recently been introduced in several family medicine units (FMUs) with a teaching mission. The objectives of this paper are to analyze the principles of advanced access implemented in FMUs and to identify which factors influenced their implementation.

**Methods:**

A multiple case study of four purposefully selected FMUs was conducted. Data included document analysis and 40 semistructured interviews with health professionals and staff. Cross-case comparison and thematic analysis were performed.

**Results:**

Three out of four FMUs implemented the key principles of advanced access at various levels. One scheduling pattern was observed: 90% of open appointment slots over three- to four-week periods and 10% of prebooked appointments. Structural and organizational factors facilitated the implementation: training of staff to support change, collective leadership, and openness to change. Conversely, family physicians practicing in multiple clinical settings, lack of team resources, turnover of clerical staff, rotation of medical residents, and management capacity were reported as major barriers to implementing the model.

**Conclusion:**

Our results call for multilevel implementation strategies to improve the design of the advanced access model in academic teaching settings.

## 1. Background

Timely access to primary healthcare continues to be a significant challenge for patients around the world. Excessive wait time for an appointment with a family physician is the subject of much policy discussion, numerous governmental reports, and substantial bodies of research worldwide. Lack of timely access to primary care has been documented among the causes of inappropriate use of emergency departments [1, 2]. Providing appropriate and timely access is a prominent health policy issue and a top priority on the healthcare reform agenda in many countries including Canada. It is considered as one of the hallmarks of a high quality healthcare system [3] and a key performance indicator in primary care used by* The Commonwealth Fund 2015 International Health Policy Survey* covering 11 countries [4]. According to this survey, Canada has made some progress in this area but still ranks 10th on a list of 11 industrialized countries. The proportion of Canadian family physicians able to provide the same or next day appointment to almost all or most of their patients when requested is significantly lower than the Commonwealth Fund's average (53% versus 72%) [4].

Among a wide range of interventions [5] (e.g., primary care teams, group medical visits) recommended to improve primary care access, advanced access is known as a patient-centered innovation [2] that has been specifically designed to offer a timely access to care. It is a promising intervention that has been promoted internationally and across Canada. Advanced access is a set of five guiding principles (balancing physician's service supply with patient demand, reducing the backlog, reviewing the appointment system, integrating interprofessional practices, and developing contingency plans) to improve the ability of patients to schedule an appointment with their primary care provider on the same or next day for any types of visit and for any problems encountered [6, 7].

Studies regarding effects of advanced access have shown improved timely access to primary care, improved practice efficiency and quality of care, and increased patient satisfaction [2, 8–13]. Although it has been the focus of many international studies, most research aimed to analyze the implementation of some key guiding principles developed by Murray and Tantau [6], on measuring appointment availability and on evaluating its effectiveness for patients, professionals, and practices.

Factors influencing implementation have been less explored despite the fact that transitioning from a traditional to an advanced access model is not without challenges [14]. Furthermore, few papers have attempted to conduct a comprehensive evaluation by using conceptual frameworks although they are known as essential tools for designing strategies to better address implementation challenges [15]. True et al. [16] conducted a formative evaluation which was informed by the Consolidated Framework for Implementation Research but narrowed the focus on readiness for implementation showing the impact of three key characteristics—leadership, staffing resources, and access to information—on implementing interventions related to advanced access.

To date, a few studies have investigated factors that influence implementation of advanced access in a primary care setting, but most took place in the United States, the United Kingdom, Australia, and some western Canadian provinces. They have mainly shown that factors such as leadership, team engagement, resources [10, 17–19], organizational support [17, 20], and attitudes of physicians [20, 21] play a key role in successful or failed implementation.

Given the variety of factors influencing the implementation of advanced access, across contexts, there is a crucial need to conduct a comprehensive evaluation based on a multilevel framework in order to provide a better understanding of how implementation efforts succeed or fail [15]. Considering that Quebec, one of Canada's worst provinces with regard to timely access, has recently introduced the advanced access model in several primary care organizations, timely evaluative information is needed. To date, only one exploratory study was conducted among early family physicians adopters showing a range of factors that may either enhance (e.g., physicians' leadership, availability of professional resources in the organization) or impede the adoption of advanced access (e.g., resource availability, team functioning) [22]. However, the results were limited to the perceptions of the early adopters of advanced access. Although family physicians are key stakeholders in the change process, research needs to give voice to each of the groups of stakeholders involved in this process to capture the complexity of the change.

This paper builds on prior work by incorporating the views of different stakeholders in family medicine units (FMUs) undergoing the implementation of the advanced access from various local networks in the universal healthcare system in Quebec, Canada. FMUs are academic teaching units essentially dedicated to training medical students and residents in primary care. They represent an opportunity to expose students and residents to new practices and to a real-life educational experience, challenges and successes that advanced access entails, which can serve as a lesson for implementation in their future practice. Thus, improving the implementation of advanced access in FMUs is a key strategy to induce a systemic effect in academic settings and to better meet patients' needs.

The main objective of this paper is to evaluate the early experiences of implementing the advanced access model in primary care units with a teaching mission (family medicine units). Specifically, we aim to analyze the principles of advanced access implemented in FMUs and to identify which factors influenced the implementation (positively or negatively) of these principles.

The results may help identify useful lessons for other FMUs or similar teaching units planning to implement the advanced access model and develop adapted strategies for implementation.

### 1.1. Framework

Our adapted framework builds on Murray and Tantau's guiding principles of advanced access [6] and Chaudoir et al.'s [23] determinant framework for implementation analysis.

#### 1.1.1. The Guiding Principles of Advanced Access

Five guiding principles of the advanced access model adapted from the original work of Murray et al. [6, 17] were included in our framework and are outlined in Table 1.

#### 1.1.2. The Chaudoir Conceptual Framework

We adopted the Chaudoir multilevel framework that accounts for a patient-level factor because we consider that it is of primary importance to assess how innovations' beneficiaries impact the success of implementing advanced access [23]. Our adapted framework stipulates that the implementation of the innovation may be influenced by a range of interrelated factors at multiple levels that should be considered when analyzing the implementation process. (1) The first factor is* the broader structural context*, where the implementing organization (FMU) is nested, such as legislative rules, regulations, funding and policy support, and interorganizational dynamics (e.g., collaborations between clinics and hospitals). (2) The second factor is* the organizational level* which is related to the clinical care settings or FMU characteristics, such as leadership and commitment of leaders to implement changes, availability of organizational resources, organizational culture, governance structure, the nature and quality of networks, and communication and information sharing among different health professionals and staff of the FMUs. (3) The third factor is* the characteristics of the users of the innovation* (e.g., professional and clerical staff in the FMUs) such as practice profile, knowledge level, and attitudes towards the model, skills, and motivation [24, 25]. (4) The fourth factor is* the characteristics of clienteles* targeted by the innovation and expected to benefit from it such as their health-related beliefs and socioeconomic and demographic profiles.

## 2. Methods

### 2.1. Research Design

A multiple qualitative case study design was used to thoroughly understand a complex phenomenon within its real-life context [26]. Four FMUs were selected to represent different geographic areas, diverse experience in terms of transitioning from traditional to advanced access model or starting up with the model, and timeframe for implementation (more than one year). They were chosen among the early adopter FMUs that are the first units undergoing the advanced access implementation process (see Table 2). We targeted a sample of FMUs with an early adopter profile because FMUs across Quebec were mainly first adopters since the advanced access training provided by Quebec's Federation of General Practitioners and the Ministry of Health and Social Services was initiated between 2011 and 2012.

### 2.2. Data Collection

Data collection took place from July 2015 to February 2016. A researcher led recruitment and data collection on each site. For each FMU, information about the project was e-mailed to medical directors who were invited to participate in the study and to appoint a key person to facilitate the recruitment procedure and to provide e-mail addresses of key informants who were actively involved in the implementation.

Information on implementation was obtained from a total of 40 semistructured interviews that were conducted with a diverse sample of primary care providers selected to ensure representation of all relevant health professional groups (family physician directors [FPD], family physicians [FP], residents in family medicine [RFM], registered nurses [RN], nurse practitioners [NP], and clerical staff [CS]). This allowed covering the broadest range of perspectives from stakeholders who drove the change and obtaining an in-depth understanding of each case. Data saturation was reached with ten key informants within each setting.

Of the 40 interviews, 21 were carried out face-to-face and 19 were conducted over the phone with participants working in distant regions. Face-to-face interviews lasted 50 to 60 minutes and telephone interviews ranged from 40 to 45 minutes. All interviews were conducted in French and representative quotations were translated by a bilingual research staff and checked by all bilingual authors for accuracy and equivalency of information translated.

Interview guides adapted to the informant's specific role were used and covered: (1) the extent of implementation of the key principles of advanced access; (2) the various factors perceived as having influenced implementation; and (3) recommendations.

Following site visits, regular meetings were held by the research team to discuss key themes emerging from interviews. Interviews were complemented by secondary data sources: guiding documents (*n* = 2) developed by two units (cases 1, 3) to implement the advanced access, documents published by the Federation of General Practitioners of Quebec, and academic reports (*n* = 2). These documents provided an insight into the local context (FMUs) and larger context regarding many issues (e.g., history and reasons for change, tools for implementing the advanced access).

Ethical approval was obtained from* Centre de Santé et de Services Sociaux-Institut Universitaire de Gériatrie de Sherbrooke*. Written, informed consent was obtained from all the participants before their participation in the interviews.

### 2.3. Data Analysis

Our framework served as a conceptual basis for assessing the implementation of the key principles and the barriers and facilitators across cases. Data were transcribed and entered into QDA Miner software (version 14). Qualitative content analysis was performed following Miles and Huberman's steps: data reduction, display into matrix, and data interpretation and conclusion drawing [27]. We used a combined deductive and inductive approach. The levels of the framework and the five core principles of advanced access formed the initial codes list. Additional codes were added based on inductive analysis of the empirical data. A detailed narrative case study report was developed for each FMU. Matrices were also constructed by category (level factor) for each case. This was followed by displaying data in cross cases matrix for analyzing similarities and differences in barriers and facilitators experienced across cases [26]. To ensure credibility of results, data and investigator triangulation were used in this study [28]. Seeking perspectives from various groups of stakeholders helped in data triangulation. A summary of results was fed back to participants in two FMUs under study, and a feedback presentation session was held in each FMU. Comments were incorporated into the final research results.

## 3. Results

We present our findings according to the study's objectives.

### 3.1. Objective 1

Objective 1 is analyzing the principles of advanced access implemented (see Table 3).

#### 3.1.1. Balancing Supply and Demand

In three FMUs (1, 2, and 4) a retrospective measure for provider supply and patient demand was undertaken for each provider of the unit's medical team. In two cases (1, 4), this essential activity was done using a team approach and conducted by the unit as a whole. It was followed by the implementation of strategies to handle demand and restore the balance between supply and demand such as redistributing workload (e.g., from physicians to nurses) and dropping certain activities.

*In fact, that's what we did: all the calculations for advanced access. I did it with each physician: I took data from the RAMQ [the Quebec Health Insurance Board] to know how many patients each physician had and then I counted how many appointments they had had in the previous year. Then after that, I met them and asked them “How many vacation days you are taking this year? How many days you do at the hospital?” Eh, it's about 150 to 200 patients per half day per physician. That's the rule! I met them all, I had young ones who had room to take [patients], had older ones who had too many, those who had too many I matched them more with nurse practitioner*. (FPD-FMU1)

In case 2, an attempt to understand and balance supply and demand was initiated by two physicians. They tried to mobilize their colleagues in the clinic by sending each physician his or her own data related to the imbalance between supply and demand. Despite supply and demand being poorly matched, family physicians were left on their own to restore the balance which led to few physicians (3/20) putting in place effective strategies such as modifying their practice to align their availability with the number of patients enrolled.

*Well, right now they [physicians] have abandoned because they have been in practice for a long time and like us, they are involved in many activities that they can't leave behind. But at least, to make them realize that, see, you are going to fail for sure, your supply is too small, so stop enrolling patients.* (FP2-FMU2)

Conversely, this activity was not applicable to case 3 given that it was a new FMU starting up with the advanced access model at the outset. However, family physicians were expected to manage their panel size on their own.

*Appointments lengths* were standardized following the implementation of advanced access in FMUs 1 and 4 to allow greater flexibility in organizing supply for patients. They ranged from 15–30 minutes for shorter appointments to 45–60 minutes for longer appointments.

Introducing methods to reduce demand for visits varied across FMUs. For example, in three FMUs, the periodic annual exam was eliminated while in one FMU (2) physicians were still trying to phase it out gradually. Patient education regarding this change was a central issue to the majority of participants interviewed.

*Yes, people come for their annual exams, but we are trying to change this slowly, not quickly; to change the mindset of patients*. (NP-FMU2)

*Maximizing activities at each appointment *was also the focus of change in three FMUs (1, 3, and 4) which tried to cover multiple issues during a single visit and pool acute care visits with routine exams (e.g., visit for acute problem and mammogram screening) for some patients and consequently reduce their return visit.

*So if you ever come for reason X that is not related to your annual exam, but you were due for a mammography, your gynecological exam or whatever, I'll do it at the same time I see you for reason X. It'll prevent you from coming twice. So we try to condense appointments*. (FP1-FMU3)

#### 3.1.2. Reducing Backlog of Scheduled Appointments

Physicians eliminated the backlog of patients in two FMUs using either a quick strategy over few months (case 1) or a progressive strategy over 6 to 12 months (case 4).

*And what was a waiting list was officially abolished. But, we had been preparing for a year and, so, there wasn't much on the waiting list*. (FPD2-FMU4)

In FMUs 2 and 3, they did not have an appointment list to be cleared out. Underlying reasons for this difference were that one FMU (3) adopted advanced access upon opening and the other (FMU 2) did not have a waiting list, with the exception of a few physicians, because this clinic had a philosophy of patients being responsible for booking their appointments.

Similarities regarding patient education for the expected change were identified. In all cases, clinics used a combination of methods, mainly letters and verbal explanation by phone and at each visit as well as reminder cards. Patient education was done over several months before and after converting to the new appointment system. One FMU also used posters in the clinic and published a notice in the local newspaper in addition to the other methods.

*We published in the local newspaper. We handed some documents, we made a lot of photocopies. We put up big posters in the waiting room. All the patients of physicians in the team who walked in received the information*. (RN3-FMU4)

Data analysis showed that two strategies were used to clear the backlog. One was to review each provider's schedule and cancel unnecessary future appointments when possible (FMUs 1, 4). The other was to provide extra appointments temporarily by adding office hours for a limited period of time (FMU1).

*We asked physicians. We handed them a list of their patients and asked them to identify those we could remove who from the list and those we should for various reasons: vulnerable patients, those who did not have a lot of support, mental health or whatever, those who were afraid of not being able to make an appointment by themselves*. (CS-FMU1)

#### 3.1.3. Reviewing the Appointment System

Three FMUs (1, 3, and 4) implemented a model that features 90% open slots over three- to four-week periods and 10% prebooked slots, that is, appointments booked more than three or four weeks in advance. In FMU 4, some family physicians had adopted a 100% open scheduling model. Within these FMUs, FMU 4 initiated a two-phase approach, a small scale pilot implementation in the beginning followed by a large scale implementation, whereas FMU 1 led a large scale change all at once.

*Because at the beginning, it was 50 percent of the unit and the other 50 percent started in January 2015*. (FP4-FMU4)

In FMU 3, they had to readjust their initial 100% open scheduling model over two-week periods, to a model with 90% open slots over four-week periods, allowing for 10% prebooked appointments; after assessing that their initial model was not achievable because, as a new clinic, they had a large demand from new patients for appointments.

*Well, sure it wasn't really working here. Because the problem we had was that we were also a new family medicine unit. When you're a new FMU, you have lots of new patients, it makes it difficult at the beginning to use advanced access when you have lots of new patients. […]. We have to take more time because there were many patients who were calling. We ended up with many patients who were calling constantly to try to get appointments as new patients because these appointments, let us say, are for one hour. One hour when you just have three hours in the morning, it means seeing just three new patients*. (FP4-FMU3)

The remaining FMU (2) attempted to implement advanced access but ended up implementing some form of the carve-out model by introducing 50% of appointment slots open for semiurgent and urgent care needs and maintaining 50% of prebooked appointments over two-month periods instead of three.

*I can't say that we're making full use of the advanced access model. I think it would be a lie if we said that we are using the advanced access model here. We are trying to work towards that, but I think that we still have a long way to go before we can say that we are working according to the advanced access model*. (FPD-FMU2)

While 90% of appointment slots remained open for patients, 3 FMUs (1, 3, and 4) booked 10% of appointments and maintained recall lists to meet the needs of patients who required regular prescheduled follow-up appointments (e.g., patients with chronic disease, pregnant women, infants, elderly patients, vulnerable patients, and patients suffering from cognitive impairments). In the remaining FMU (2), a follow-up list and a reminder system managed by front line staff were deemed to be necessary measures to introduce in the future. The aim of these measures would be to address the insecurity that might be felt by patients and to prevent losing track of those unable to make their own appointments when needed.

*But there are some elderly clients, the vulnerable, if we are going towards a system where we really reduce the time for our appointments to two or three weeks, we will have to have a reminder to these patients then, because they'll feel insecure and they'll be afraid of not being able to get an appointment*. (FPD-FMU2)

#### 3.1.4. Integrating Interprofessional Practices

Although interprofessional collaborative practice had already been established in the four FMUs under study, participants reported that advanced access reinforced the collaboration between physicians, nurses, nurse practitioners, and clerical staff in two units (1, 4).

Our data showed that two FMUs (1, 4) optimized resource utilization through implementing a joint practice model for a panel of patients while it was not done for cases 2 and 3. In case 2, the change was only conducted by a few physicians and nurses were not involved in the implementation process, whereas in case 3, it was not applicable given that one nurse was assigned for the whole FMU.

Our data showed that advanced access led to the development of team-based care, through establishing two new group practice models: a joint nurse/physician practice model in FMU 1 and a small team configuration in FMU 4. For example, pairing a group of four physicians with one nurse or/nurse practitioner in FMU 1 allowed nurses and physicians working in partnership, to jointly look after their patients' needs and carry out alternate or simultaneous coordinated follow-up visits for a diverse clientele such as patients who were pregnant or patients with chronic illness. In FMU 4, the creation of a small team configuration composed of a mixture of four physicians, two residents, a registered nurse, a nurse practitioner, and a clerical staff member facilitated the follow-up by the team for all types of patients.

*We are divided into teams with clerical staff in the family medicine unit and my physician partners are in the same team as me*. (NP1-FMU4)

This change led to expanding nurses' roles and optimizing their practice to a fuller scope including pregnancy follow-up, caring for patients with mental illness, and assessment of asymptomatic patients or other clienteles. As reported by a nurse,

*depression, anxiety, mental health, I am seeing these patients now and I wasn't before. I think there is a great need in this area. Patients with chronic pain, the physician refers them to us to ensure that there is a good progress. We make the adjustments depending on the needs, while consulting the physician*. (RN2-FMU1)

By contrast, two FMUs (2, 3) did not implement such strategies and consequently nurses' roles were not subject to change in these units. Nevertheless, one unit (2) expressed the desire to make future change and was exploring ways to put in place new collaborative practice models and to enhance nurses' roles in the team, while another (3) was seeking additional nursing staff to be able to make such change.

*Well, for the time being nurses are working to reorganize the way they work in order to have nurses paired with a team of physicians*. (FPD-FMU2)

Regarding redesigning the role of the clerical staff, all units considered this as key element to implementing advanced access. However, training to help the clerical staff develop the necessary skills for managing patients' requests for appointments and referring patients to the appropriate provider was instituted in only two units (1, 4) whereas they were still at the adjustment phase in FMU 3 and at the early stages in FMU 2.

They have started to ask questions, because they see the semi-urgent slots in two months, “Is it for a semi-urgent appointment or for an appointment that is not urgent?” This is the kind of question they started asking. But it is still in early stages. (FP2-FMU2)

#### 3.1.5. Creating Contingency Plans

Three FMUs (1, 3, and 4) have created formal contingency plans for physicians' absence and one FMU (1) also created a plan for nurses' absence while the remaining site (FMU 2) relied on informal arrangements system. Formal contingency plans were easily introduced in FMUs 1 and 4 using a team-based approach to care. For example, through the joint practice nurse/physician model in FMU 1, the nurse or the nurse practitioner could substitute for the physician-partner during his absence and vice versa and see patients within the core-team's patient panel such as those with chronic illness, acute medical problems, pregnancy follow-up, or other clienteles. In the physician's absence, the nurses can also seek assistance from other physicians in the unit who can support them when a clinical case is beyond their competence or when needed, whereas, in FMU 4, the creation of a small team configuration allowed team members to provide cross-coverage during periods of absence.

*So depending on the procedure, it could happen that the doctor is not here for three weeks, so I will see if another doctor from the configuration team is present, who could take the patient. I could just get him to talk with a nurse, who can see him if it concurs with her competencies, and the patient could get his problem resolved, or else he might see the nurse practitioner, to say something, or a resident, or doctor belonging to the concerned configuration […]. But that means that the configuration has almost a total around 2,000 patients. So everybody is responsible for these 2,000 patients*. (RN4-FMU4)

As for FMU 3, to cover for absent providers, it adopted a strategy of patients being seen by the same type of health professional whenever possible or, if no professional of the same type is available, by another team member.

*But for sure if a patient has a resident family doctor and there is no other resident who could see the patient, then we try as a last resort to refer to a staff doctor, so the patient can be seen by someone. We really try to keep it this way doctor-doctor, residents all together, and nurses the same thing*. (CS-FMU3)

The remaining FMU (2) relied more on informal arrangements between professionals to cover for absent colleagues and also on diverting patients to the unit's walk-in clinic. As for medical residents, in all FMUs, they informally arranged to cover for each other during off-site rotations and in some cases nurses substituted for them on request by following their patients during their absence.

Another contingency plan (FMU 1) involved pre- and postvacation scheduling, increasing and extending working hours before leaving on vacation and upon returning to the unit to meet demand.

### 3.2. Objective 2

Objective 2 is identifying which factors influenced the implementation of the principles of advanced access.

Analysis has shown 9 pivotal themes characterizing advanced access sites which facilitated or hindered implementation at the structural, organizational, professional, and patient levels (see Table 4).

#### 3.2.1. Structural Level

*Advanced Access Training*. The training sessions offered by the family physicians' association were described unanimously by participants as beneficial to implementation in all four sites. The value of training workshops to understand the underlying philosophy and rationale for the advanced access model was frequently mentioned.

*The workshop provided by the FMOQ [Quebec's Federation of General Practitioners]. Well, that's the model we got […]. It was extremely useful. Extremely. For me anyway, it was really our guide*. (FP4-FMU4)

Providing support through training tool was, according to interviewees, essential to gaining the skills regarding how to balance the supply and demand and convert the practice to this new model. Attending training sessions was also a learning opportunity and an effective mean to spread lessons learned to other health professionals in the unit who did not benefit from the training.

*Physicians' Practice and Rotations in Multiple Clinical Settings*. One of the common barriers reported across the four sites was the lack of physician availability in FMUs due to the presence of multiple part-time physicians who are engaged in diversified practices. This can be explained by Quebec's coercive policy that forces physicians, based on their years of work experience (less than 20 years), to dedicate a portion of their time (12 hours/week) to performing a number of specific medical activities in different clinical practice areas (e.g., hospital, emergency, and administrative duties). Consequently, this mismatch between providers' panel size and clinical time decreases the physicians' capacity to meet their patients' demand and to work down their backlog.

With regard to medical residents, a major influencing factor was their mandatory out-off-office rotation, required in their residency program to gain learning experiences, in a range of practice settings (e.g., hospital-based rotations, geriatrics, and rural practice). This results in residents attending to their patients only one-half day per week throughout their training, which compromises their ability to provide prompt appointments and adequate daily access to their patients.

*Well, the schedule, I would say it's more constraining in the sense that if you're ever on call at the hospital or on call in obstetrics or in the emergency, well, sometimes it's about a week's period in which we are not available. So, patients can still be directed to the walk-in clinic, but they are not seen by their doctor. So, I would say it's still a little bit limiting*. (R2-FMU4)

#### 3.2.2. Organizational Level

*Leadership*. Two FMUs were distinctive in the leadership strategy used to conduct the change. Collective leadership (executing implementation by a committed champion adopting a teamwork and work distribution approach to change) in two FMUs (1, 4) emerged as a key facilitator to implementing advanced access whereas cases 2 and 3 lacked this powerful driver. In the latter, implementation relied mainly on two physicians who failed to lead the change through collaborative efforts, to develop a collective teamwork approach and share responsibility among staff.

Engaging the whole team in FMUs 1 and 4, early on and in all phases of the process, establishing an open communication plan through regular team meetings, giving timely feedback to the entire staff, and coconstructing access improvement strategies helped put the entire staff on the same page and move forward in the implementation. This helped to create a shared vision, to have a unified goal that aligned team members, and to facilitate the introduction of processes related to advanced access.

*It has been a long process anyway. We have sat down all together, so doctors, nurses, NP…. So, it was too much work, a lot of meetings, brainstorming sessions, bringing out ideas, and what is our main goal. For us, it was about addressing the needs of patients, so we tried to see what their needs were. We have made a list of reasons for consulting. After that, we thought about who could do what*. (NP1-FMU1)

*After the training, the rest of the team was invited to join the implementation follow-up meetings. It was meetings that were held, […], regularly, every two weeks. During lunch time, we used to meet, doctors, nurses, secretaries, other professionals, progressively we placed the basics, the project milestones and were making the required adjustments and also for planning and implementation, so it was going pretty well*. (RN2-FMU4)

In contrast to these FMUs, case 2 was characterized by a lack of common vision among staff as well as a lack of active engagement of nurses and administrative staff in the process. Moreover, unequal training and lack or fragmented knowledge sharing regarding advanced access, mainly with nurses, clerical staff, and residents, were reported.

*Yes, it is possible to train people. And by doing something a bit more collective, I have a feeling that - you know, there are two people who have been there, after that three people, the fact that it is a little more fragmented, maybe it's less a collective movement. There may also be the fact that if we do not speak later on, but well maybe if it had been a while since it was started, meetings and all, but we have never heard about it, perhaps communication was lacking somewhere*. (RN-FMU2)

After encountering problems related to a lack of trained clerical staff in the postimplementation phase, FMU 3 put in place a reactive strategy of adjusting the procedures, providing a training session to adequately train the clerical staff, and elaborating a tool to guide them in their role.

*So, it's true that during the first period it was difficult at the level of clerical staff. They quickly came to see me because they did not know exactly how to direct the patients and the physicians couldn't stop talking about advanced access, but they were not familiar with it. So, I had to do a small briefing with physicians, with secretaries to explain things*. (RN-FMU3)

Participants from both sites consistently stressed the need to have a committed leader who actively engage the whole team in a collaborative change process from the early stages.

*Resources*. Three FMUs (1, 2, and 3) faced important barriers related to different types of resources. Cases 2 and 3 experienced mainly shortage of physicians, but also nurses, due to maternity leave or retirement. This led to problems in matching the supply of clinicians to patients' demand. Insufficient number of clerical staff (cases 1, 3) led to a poor management of the appointment system and made reaching the unit by phone more difficult for patients. Also, lack of adequately trained staff was overwhelmingly regarded as barrier to implementing advanced access in two FMUs among various categories of professionals. Trained clerical staff were perceived as crucial as they play a pivotal role in the process of change.

Increasing or providing additional staff was reported by these FMUs as crucial to match the demand to the supply, to improve ease of reaching the clinic by phone, to enhance interdisciplinary team work through implementing a joint practice model, and to improve accessibility. High turnover rate of clerical staff was also perceived as a serious threat to the implementation of advanced access. It negatively influenced their level of understanding of the new scheduling system and the appropriate use of the formalized algorithm to determine the nature of patients' calls and referrals to the adequate professional. Many interviewees from FMU 3 spoke about how difficult it was to adapt to staffing changes and expressed their concern and frustration about the instability of the clerical staff. One medical director mentioned that the turnover of clerical staff represents an ongoing challenge and explained how they were wasting time training new clerical staff who only stay in the unit for a few months.

*Well, this is another problem…because at the moment, our clerical staff, they are replacing…So, we had staff replacing other staff one following the other; which means training all the staff each time, it's really difficult. Right now, I don't know if the clerical staff that we have…if it's her permanent position or is just replacing. The two staff members that we have, have been working here for two to three months. I assisted them for two hours in the beginning of January, to make sure during their stay that they understood well. But […] it's a lot of energy, to train, and the core principle of advanced access is that it should run quite well when you have clerical staff who are knowledgeable about advanced access, who know how to refer patients…. I could invest some more time and meet with them every two to three weeks, but next month, maybe they won't be here anymore, so at some point, I am not using my time properly either.* (FP3-FMU3)

Dedicating full equivalent time resources, and providing well and equally trained health professionals and clerical staff, was cited as a crucial step in the early phases of implementation.

*Management Capacity*. Management capacity was identified as a barrier to implementation in three FMUs (1, 2, and 3). Working in clinics such as FMUs in Quebec limited the management capacity of clinic administrators and medical directors in terms of decision-making power and capacity to recruit and allocate sufficient and adequate professional, technological, and even financial resources to implement advanced access. While management capacity would theoretically be a facilitating factor, in the context of Quebec, this capacity is a barrier to hiring and selecting qualified clerical staff with the adequate competencies to work in this new scheduling model—one FMU (3) reported being imposed secretaries without medical background—and choosing appropriate electronic medical tools that fit the FMU's needs which were issues raised by many informants. It should be noted that, in local community health centers, hiring of a new secretary to work in advanced access is impeded by union contracts based measures such as seniority.

*Formalized Tool*. The elaboration and use of unified and standardized tools (e.g., algorithm, document) within three FMUs (1, 3, and 4) facilitated implementation in many ways. Many participants vividly stated that this valuable tool helped to clarify the team members' roles, to determine their professional boundaries in relation to advanced access, and to clearly outline the rules and steps to be followed by the clerical staff in order to provide a timely response to patients' needs. It was described as a major facilitator for accurately assessing, prioritizing, and addressing the patient's concerns and for referring them to the appropriate professional within an adequate period of time.

*They have an algorithm that mentions, well, according to the type of request, they see what the first ideal resource is for this patient who can respond to his need. And then, when they see that this resource is not available, let's say his family physician then on the algorithm that clearly shows a second option. So it can be the nurse practitioner or a clinical nurse or a resident doctor who would have an available slot. So, the algorithm shows three options for each reason of consultation, the staff just follows it eventually. They follow the numbers; for a reason, it will go to number 1, “Well, it is not available, I'll go to number 2, available, yes, no, or I'm going to number 3.” It is a work tool for our administrative staff*. (CS-FMU1)

It was obvious in our data that it helped coordinate activities and facilitate collaborative work between team members. In the remaining FMU (2), participants acknowledged the importance of developing an algorithm in the future to facilitate the implementation of advanced access.

*Openness to Change*. The different members' openness to change across three FMUs was interestingly highlighted as facilitating the implementation of advanced access. Participants met and expressed their openness to a full collaborative and interprofessional practice model incorporating an enhanced nursing role including sharing responsibility for patients. For example, despite the fact that FMU 2 was still trying to drive the change to implement advanced access, participants were enthusiastic about enhancing collaborative practice. For instance, discussions had started to take place about which collaborative practice model to set up between nurses and physicians and about how to align their expertise with patients' needs. Also, the ability of team members to tolerate uncertainty and considering errors as opportunities for learning and their openness to knowledge sharing and expertise exchange were reported as facilitating learning for nurses and contributing to the development of administrative and nursing staff roles.

*It's about being open, all key stakeholders. The secretary, clerical staff, physicians, nurses, supervisors, we need everyone to be open to collaboration and to trust each other. That's the key, being open to change. The ability to accept uncertainty too. The capacity to tolerate uncertainty is one of the keys because we are not good right away when performing new practices, they can make mistakes. You must trust…. It takes the flexibility. This is the opposite of rigidity. If you're rigid and you need protocols, it doesn't work because here we do pap tests. Aye, it is no good, we start again*. (RN1-FMU1)

#### 3.2.3. Professional Level

*Resistance to Change*. Within FMUs 2, 3, and 4, some physicians showed uncooperative attitudes particularly in the early stages of implementation. FMU1 was the only unit where this factor was not mentioned as a major issue. Reluctance to embrace change was related mainly to practice culture and mechanisms, to lack of understanding the practice philosophy of scheduling, to lack of availability of physicians in the unit, and finally to fear of loss of patients. Also, a number of comments highlighted that older physicians tended to resist change more than younger physicians. In FMU 3, one member of the clerical staff mentioned that there was a tendency among some physicians to revert back to the old system due to difficulties in giving up preconceived notions from the traditional system.

*Well, there are some physicians who, if the same day we don't have a patient to put in an advanced access slot, will decide to see a patient, a new patient instead. So instead of keeping their slots available in case someone calls, if by noon they see there is no patient, they will ask us to find a patient to add in order to complete their schedule […] When there is no patient, there are physicians who are not happy*. (CS-FMU3)

#### 3.2.4. Patient Level

*Patients' Culture*. Changing the mindset of patients who are accustomed to the traditional scheduling system and convincing them to give up their old habits regarding prebooked appointments and annual physical examination were highlighted as critical barriers in the initial phase of the implementation. It was even considered as an ongoing issue in three FMUs.

*The barrier is the culture, changing the culture. As I said, patients are used to acting a certain way and are not necessarily curious to our change, and they just need to call when they feel the need to be seen and they will be seen*. (CS-FMU1)

Patients had difficulty understanding that, with the implementation of advanced access, they could now promptly access their physician (e.g., same day or next day appointments, appointments within the next two weeks depending on their needs) and no longer needed to book appointments more than two weeks ahead of time or to go to emergency room.

*Surely, there is here a culture like going to emergency room when you suffer from sinusitis. We are working on this issue, but it's a culture very embedded in the context […]. Most people have the reflex of going to the emergency when they have an acute problem. But slowly, it's changing […]. Many people don't think that it works, that they can call and get a prompt appointment. It will take few years before they get used to it*. (FP-FMU4)

Patients' culture regarding nurses' competencies and ability to perform tasks traditionally done by their physician was also a barrier in FMU 1. Continuous education of patients to remind them repeatedly about the change, by a variety of measures such as verbal explanation, reminder cards, and the phone was a common recommendation which participants emphasized as being essential to improving implementation.

## 4. Discussion 

This study is the first to investigate the implementation of advanced access in family medicine units in Quebec. Our results show that FMUs have variable levels of operationalization of advanced access and have implemented different combinations of its key principles. Two FMUs adopted the majority of the guiding principles recommended by Murray and Tantau [6] whereas one FMU has introduced only some of the principles (e.g., revamping the scheduling system and developing contingency plans). The remaining FMU has made some attempts to improve access to care but failed to implement core principles of advanced access. The latter has focused on redesigning the scheduling system and placed less emphasis on changing particular principles (e.g., integrating an interprofessional practice, expanding nursing roles) despite the fact that it is critical for effective implementation of advanced access. Indeed, optimal integration of nonphysician health professionals such as nurses is often overlooked when implementing advanced access [29] despite the fact that it is known as one of the key-high levers for changes of the model and a powerful strategy to eliminate wasteful delays [14, 30] and to address challenges such as lack of accessibility and continuity of care. One possible reason may be a limited understanding of the model of advanced access and how to assess the clinic's readiness for such change, which both need to be addressed as documented in other studies [18, 30].

Our results clearly show that an interplay of complex factors falling within the five nested levels of Chaudoir's framework contributed to facilitating or impeding implementation.

Despite variation in implementation levels, many barriers and facilitators to implementing advanced access were common across the FMUs regarding the structural, professional, and patient levels. Our results did not show a clear pattern of influencing factors in terms of levels of implementation. However, two barriers (leadership, availability of human resources) that distinguished between low and high levels of compliance with the key principles were related to the organizational level.

*At the structural level*, the influencing factors were similarly important across all units.

One notable barrier to continuity of care is the practice setting that warrants particular attention. Continuity could be impeded due to the irregularity of provider availability in academic units as already reported in other studies [31, 32]. Our findings suggest that a team-based approach through which patients are cared for by a team of health professionals well informed about their case is a promising solution to ensuring continuity and providing timely access at the same time. They corroborate the recommendations of many authors including the concept's founder [10, 22, 33] to choose team-based continuity rather than an individual physician to avoid waiting times in academic unit settings.

Our results show also that the formal training offered* at the structural level* is an important facilitating factor but does not guarantee the implementation of advanced access at the unit level. On its own, it is unlikely to drive change if there is a lack of a key organizational factor, leadership in terms of increasing the organization's capacity for learning and change through sharing the collective experiences of its members, and focusing on collective achievement [34, 35].

Despite the influential effect of external factors, our results suggest that two factors at the organizational level (leadership strategy, availability of human resources: nurses, physicians) seem to be related to the level of implementation and crucial to implementation success or failure. For example, collective leadership appears to be a dominant factor and a key driver to successful implementation acting on many levels (e.g., the professional level, outside and inside the organization). In fact, FMUs that had implemented the majority of the key principles had a common and significant facilitating factor, that is, leadership strategy. Our data shows that a clinical champion leading the implementation on his own or with few physicians was not sufficient, as has been highlighted in some studies [9, 36]. Overall, units that did not embrace a collective approach nor promote a shared responsibility for implementation compared to those which did failed to implement some key principles (integrating an interprofessional practice through implementing a joint practice model, transforming nurses' roles, and developing contingency plan at the unit level). Those units did not succeed to engage the whole staff (e.g., nurses) in the change process, to achieve internal organizational alignment and support, equal information sharing, coaching and training of all staff, and a common understanding of advanced access among the clinic's staff.

Prior research has pointed to the importance of leadership (i.e., instituting teamwork [9, 37], to engage the whole staff early on and throughout the entire process) for implementation without broadening the discussion on this crucial factor [10, 19, 38]. Running two practice models in a single clinic or conducting a gradual implementation among some providers instead of a practice-wide change has also been mentioned in previous research as a source of confusion for patients and frustration for the whole staff [10, 39].

The data also revealed that poor implementation of some key principles (achieving a balance between supply and demand, integrating an interprofessional practice through optimizing nurses' roles) was also related to inadequate staffing (e.g., shortage of physicians and nurses). Lack of or fewer staffing resources have been identified as a universal barrier by teams implementing advanced access as no scheduling system can work adequately if the demand exceeds capacity, the physician and nurses supply [16, 18, 19]. Our results corroborate previous findings and highlight the importance of devoting sufficient, stable, and skilled staffing resources prior to and throughout the process to ensure that units are innovation-ready to implementing the new model and that results are sustainable [16, 40].

Other barriers such as management capacity, which was closely linked to adequacy and stability of resources, should be taken into account as it affects largely the skills and knowledge needed (e.g., clerical staff) to adhere to the new model in terms of patient management (e.g., assessing and distributing requests according to staff's availability) [22]. It should be the subject of frequent negotiations and shifts of power between the organizations bound by protective union contracts based on seniority or other measures and medical directors given the growing need to implement advanced access in public clinics (FMUs) in Quebec. In addition to the factors found in the literature, our results show that the use of a formalized tool is a facilitating factor for role redesign and collaborative practice.

*Finally, regarding the clinical staff*, our results show that implementing advanced access defied their beliefs about the scheduling system and led to attitudes of resistance, particularly when the concept of advanced access was not adequately and equally understood among the clinic's staff. Many authors have emphasized how these beliefs affect the move from the traditional paradigm of managing scheduling to the paradigm of reengineering patient care delivery. In fact, implementing advanced access requires a radical shift in thinking and behavior at the professional and unit levels [41–43]. Also, rooted habits and culture of patients constrained implementation of advanced access as they have been used to wait for long time (weeks and even months) to get an appointment and according to Murray [10] major changes can be shocking.

Suggestions made by participants regarding overcoming these barriers by providing training and education to all team members and patients throughout the process and by involving both as active partners in planning and conducting the change mirror the majority of studies' findings on advanced access [10, 22, 29, 44].

### 4.1. Implications

Our findings could have important implications for other primary academic healthcare practices considering future implementation of advanced access. They can guide policy makers and providers interested in the advanced access model in addressing these influential factors before and during implementation to increase its chances of successful implementation and improve access to primary healthcare for patients.

They concur with the recommendations of considering advanced access as a multicomponent strategy and a comprehensive innovative approach to redesigning care processes rather than a simple scheduling system [10, 37, 45]. Practices that solely focus on some principles could miss the right path to improving access.

Implementing advanced access is a complex process that requires taking into consideration a variety of multilevel critical factors. To ensure successful implementation and to increase the likelihood of sustaining the change, key strategies should be developed and adapted to the identified barriers which fit each practice's needs. Based on our data and the recommendations of many studies, an array of multifaceted implementation strategies that exert their effects at multiple levels of the implementation context may be needed [46] such as providing ongoing training to all team members and devoting sufficient time to educate patients [43, 47], adopting a team approach including nurses and clerical staff in the planning phase and during implementation [10, 20, 44] and engaging the whole team in the process within a learning environment (e.g., critical reflection on problem-solving), monitoring progress, and ensuring ongoing feedback mechanisms [36, 48]. As a consequence, further research is required to evaluate their effects.

Some limitations should be mentioned. Interviews were conducted by four different investigators which may have led to lack of consistency in data collection. However, all team members had a very good conceptual knowledge of the key principles of the model and regular meetings were held during the empirical field work to reduce potential biases. It is noteworthy that the patients' perspectives were central to the process and are considered in a separate paper.

Also, as advanced access is still unfolding and is evolving over time, it would be interesting in the future to conduct a longitudinal study to monitor changes throughout the process and to broaden our understanding of how each clinic adapts its implementation strategy and sustains the change.

Our results cannot necessarily be generalized to other primary care organizations characterized by their own pitfalls and local contexts.

## 5. Conclusion

With the growing interest of spreading this promising model in Quebec and in many countries facing the challenge of ensuring timely access to primary healthcare, our results highlight the complexity of the factors (structural, organizational, professional, and patient) that should be considered when implementing this model. Although all factors should be a key priority for effective implementation, paying particular attention to key organizational factors (resources, leadership, and team work approach) increases the likelihood of achieving successful implementation of advanced access.

## Figures and Tables

**Table 1:** The key principles of advanced access adapted from Murray and Berwick (2003) and Breton et al. (2016).

Key principles of advanced access	Definitions
(1) Balance supply and demand	To assess and understand, on one hand, the actual patient demand for appointments per physician per day, weighted by patient status and, on the other hand, the supply (e.g., number of appointments offered) in order to achieve the right balance between the two and match the demand to supply. Strategies to decrease demand for visits (e.g., max pack, extending visit intervals) or to increase supply (e.g., redesigning doctors scheduling system) are used.

(2) Reduce the backlog	To eliminate the previously scheduled appointments (wait list) through many strategies such as adding resources, increasing the supply of visits during a period of time. Communication strategies must also be put in place to inform and educate patients about the new advanced access model.

(3) Review the appointment system	To plan the physicians' schedules over a short term (two to four weeks) and smooth out the demand for visits in order to offer same day appointments for acute and urgent cases.

(4) Integrate interprofessional practices	To develop or enhance the interprofessional practice between physicians and other healthcare professionals (e.g., nurses). Professional roles need to be optimized and tasks need to be clarified to meet patients' needs in a timely manner.

(5) Create contingency plans	To plan for seasonal increases in demand and to develop coverage plans for replacing medical staff or other healthcare professionals on vacations and during illness periods. Many strategies are applied such as increasing the number of slots prior to leave and after returning on duty, hiring temporary providers, distributing and matching staffing competencies to demand. Integrating the collaborative and interprofessional practice facilitates planning for periods of absence.

**Table 2:** Summarizing the characteristics of the selected family medicine units.

	FMU 1	FMU 2	FMU 3	FMU 4
Setting	Urban	Urban	Urban	Rural
IUHSSC	South central part of the island of Montreal	The National Capital region	The Laurentians	The North coast
Team composition				
Family physicians	33	20	13	15
Residents 1st, 2nd year (R1-R2)	25	24	13	14
Advanced practice nurse	2	1	1	1
Registered nurse	4	4	1	2
Clerical staff	4		2	4
Registered patients	11000	10,000	<6,000	6700
Patient population served	All types, ages (pediatric, pregnant women, young families, elderly, vulnerable patients, etc.)	All types, ages	All types, ages	All types, ages

FMU = family medicine unit; IUHSSC = Integrated University Health and Social Services Center; R1 = first year of residency; R2 = Second year of residency.

**Table 3:** An overview of the key principles of advanced access implemented across the four family medicine units.

	FMU 1	FMU 2	FMU 3	FMU 4
(1) Balance supply and demand				
Measure provider's supply	√	√		√
Measure demand	√	√		√
Standardize appointment length	√			√
Restore balance with various strategies	√	+/−		√
Eliminate annual exam	√	+/−	√	√
Max-pack visits	√		√	√
(2) Eliminate backlog	√			√
Cancel unnecessary appointments	√			√
Provide extra appointments temporarily; add office hours for a period of time	√			
Patient education strategy				
Provide verbal explanation	√	√	√	√
Send letters to patients	√			√
Put up posters				√
Publish a notice in a local journal				√
(3) Review the appointment system				
Appointment model: 90-10% 90% open slots over three- to four-week periods and 10% prebooked slots	√		√	√
Some form of the carve-out model: 50% open for semiurgent and urgent care needs, 50% prebooked slots		√		
Maintain recall list (patients with chronic disease, pregnant women, infants, elderly and vulnerable patients, etc.)	√		√	√
(4) Integrating interprofessional practices				
Reinforce the collaboration between physicians, nurses, advanced practice nurses, and clerical staff	√			√
Implement a joint nurse/physician practice model	√			
Implement a small team configuration				√
Expand nurses' role	√			√
Redesign clerical staff role	√	+/−	+/−	√
(5) Create contingency plan				
Formal contingency plan	√		√	√
Cross-coverage within a team-based approach	√			√
Coverage for the absent provider by peers			√	
Informal arrangements system between professionals to cover for absent colleagues		√		
Informal arrangement between residents to cover for each other	√	√	√	√
Pre- and postvacation scheduling: increase and extend working hours before leaving on vacation and when returning to the unit	√			

FMU = family medicine unit; √ = strategy used; +/− = attempt to use the strategy (early stage of reflection and use).

**Table 4:** A distribution of the themes across the four family medicine units.

	Influencing factors			
Themes-subthemes	FMU 1	FMU 2	FMU 3	FMU 4
Structural level				
*Training (FMOQ, other)*	+	+	+	+
(i) Better understanding of the philosophy of advanced access matching supply to demand				
(ii) Dissemination of knowledge to team members				
*Physicians' practice and rotations in multiple clinical settings*	−	−	−	−
(i) Lack of availability of providers				
(ii) Decreased capacity to meet patients' needs				
Organizational level				
*Collective leadership *	+	−	−	+
(i) Presence of a local champion and collective approach to change				
(ii) Training and coaching of all team members				
(iii) Communication strategy and regular feedback				
(iv) Coconstruction of tools				
(v) Team development of an adjustment strategy for resolving problems encountered				
(vi) Ongoing staff motivation				
*Resources*	−	−	−	
(i) Insufficient numbers of family physicians and nurses				
(ii) Insufficient number of clerical staff members				
(iii) Lack of adequately trained professionals: physicians, nurses, clerical staff				
(iv) High turnover of clerical staff				
(v) Technology resources: dysfunctional computer system				
*Formalized tool*	+	+/−	+	+
(i) Clarifies role				
(ii) Facilitates assessment of the request and referral to the appropriate professional				
*Management capacity*	−	−	−	
(i) Lack of decision-making power: selecting, recruiting clerical staff, technology resources, allocation of financial resources				
*Openness to change*	+	+		+
(i) Nursing skill development				
(ii) Reorganizing and improving practice and access				
(iii) Exchanging expertise and interprofessional practice				
Professional level				
*Attitudes of resistance at the initial phase (physicians)*		−	−	−
(i) Misunderstanding/erroneous understanding of the concept advanced access				
(ii) Not being convinced about its usefulness				
(iii) Fear of loss of patients				
(iv) Lack of regular availability at the clinic				
Patient level				
*Culture, habits*	−		−	−
(i) Patients' responsibility for booking appointments and follow-ups				
(ii) Annual physical exam				
(iii) Patient habits (elderly patients used to book appointments in advance)				
(iv) Consulting a family physician versus going to emergency room				
(v) Follow-up visits with the nurse instead of the physician				

FMU = family medicine unit; FMOQ = Quebec's Federation of General Practitioners; factors that positively influence implementation (facilitator) = +; factors that negatively influence implementation (barrier) = −; factor that still is in its development stage = +/−.

## References

[1] Pineault R., Tousignant P., Roberge D. (2005). Research Collective on the Organization of Primary Care Services in Québec-Summary Report. *Agence de développement de réseaux locaux de services de santé et de services sociaux de Montréal*.

[2] Hudec J. C., MacDougall S., Rankin E. (2010). Advanced access appointments: effects on family physician satisfaction, physicians' office income, and emergency department use. *Canadian Family Physician*.

[3] Salisbury C., Montgomery A. A., Simons L., Sampson F. (57). Impact of advanced access on access, workload, and continuity: controlled before-and-after and simulated-patient study. *British Journal of General Practice*.

[4] How Canada Compares.

[5] Bodenheimer T., Majeed A., Bindman A. B. (2003). Innovations in primary care in the United States Commentary: What can primary care in the United States learn from the United Kingdom?. *BMJ*.

[6] Murray M., Tantau C. (2000). Same-day appointments: exploding the access paradigm. *Family Practice Management*.

[7] Knight A. W., Padgett J., George B., Datoo M. R. (2005). Reduced waiting times for the GP: two examples of "advanced access" in Australia. *Medical Journal of Australia*.

[8] Bundy D. G., Randolph G. D., Murray M., Anderson J., Margolis P. A. (2005). Open access in primary care: results of a North Carolina pilot project. *Pediatrics*.

[9] Mehrotra A., Keehl-Markowitz L., Ayanian J. Z. (2008). Implementing open-access scheduling of visits in primary care practices: A cautionary tale. *Annals of Internal Medicine*.

[10] Murray M. (2005). Answers to your questions about same-day scheduling. *Family Practice Management*.

[11] Fournier J., Heale R., Rietze L. L. (2012). I can't wait: advanced access decreases wait times in primary healthcare. *Healthcare Quarterly*.

[12] Fournier J., Rainville A., Ingram J., Heale R. (2015). Implementation of an advanced access scheduling system in primary healthcare: one clinic's experience. *Healthcare Quarterly*.

[13] (2012). *Family Physicians of Canada. Best Advice-Timely Access to Appointments in Family Practice*.

[14] Kiran T., O'Brien P. (2015). Challenge of same-day access in primary care. *Canadian Family Physician*.

[15] Nilsen P. (2015). Making sense of implementation theories, models and frameworks. *Implementation Science*.

[16] True G., Butler A. E., Lamparska B. G., Lempa M. L., Shea J. A., Werner R. M. (2013). Open access in the patient-centered medical home: lessons from the veterans health administration. *Journal of General Internal Medicine*.

[17] Murray M., Berwick D. M. (2003). Advanced Access: Reducing Waiting and Delays in Primary Care. *Journal of the American Medical Association*.

[18] Pickin M., O'Cathain A., Sampson F. C., Dixon S. (2004). Evaluation of advanced access in the national primary care collaborative. *British Journal of General Practice*.

[19] Pierdon S., Charles T., McKinley K., Myers L. (2004). Implementing advanced access in a group practice network. *Family Practice Management*.

[20] Cameron S., Sadler L., Lawson B. (2010). Adoption of open-access scheduling in an academic family practice. *Canadian Family Physician*.

[21] Murray M., Bodenheimer T., Rittenhouse D., Grumbach K. (2003). Improving Timely Access to Primary Care: Case Studies of the Advanced Access Model. *Journal of the American Medical Association*.

[22] Breton M., Maillet L., Paré I., Abou Malham S., Touati N. (2016). Perceptions of the first family physicians to adopt advanced access in the province of Quebec, Canada. *The International Journal of Health Planning and Management*.

[23] Chaudoir S. R., Dugan A. G., Barr C. H. (2013). Measuring factors affecting implementation of health innovations: a systematic review of structural, organizational, provider, patient, and innovation level measures. *Implementation Science*.

[24] Damschroder L. J., Aron D. C., Keith R. E., Kirsh S. R., Alexander J. A., Lowery J. C. (2009). Fostering implementation of health services research findings into practice: a consolidated framework for advancing implementation science. *Implementation Science*.

[25] Breton M., Brousselle A., Boivin A. (2014). Evaluation of the implementation of centralized waiting lists for patients without a family physician and their effects across the province of Quebec. *Implementation Science*.

[26] Yin R. K. (2009). *Case Study Research: Designs and Methods*.

[27] Miles M. B., Huberman M. A. (2003). *Analyse Des Données Qualitatives*.

[28] Patton M. Q. (2002). *Qualitative Research & Evaluation Methods*.

[29] Goodall S., Montgomery A., Banks J. (2006). Implementation of Advanced Access in general practice: postal survey of practices. *British Journal of General Practice*.

[30] Tantau C. (2009). Accessing patient-centered care using the advanced access model. *Journal of Ambulatory Care Management*.

[31] Phan K., Brown S. R. (2009). Decreased continuity in a residency clinic: a consequence of open access scheduling. *Family Medicine*.

[32] Baxley E. G., Weir S. (2009). Advanced access in academic settings: definitional challenges. *Annals of Family Medicine*.

[33] Mainous III A. G., Salisbury C. (2009). Advanced access, open access, and continuity of care: should we enforce continuity?. *Family Medicine*.

[34] Champagne F., Lemieux-Charles L., Duranceau M.-F., MacKean G., Reay T. (2014). Organizational impact of evidence-informed decision making training initiatives: a case study comparison of two approaches. *Implementation Science*.

[35] Chirichello M. (2004). Collective leadership: reinventing the principalship. *Kappa Delta Pi Record*.

[36] Witt M. J. (2006). Advanced access works! improved patient satisfaction, access, and P4P scores. *Journal of Medical Practice Management*.

[37] van der Voort M. R., van Wijngaarden J., Janssen S., Berden B., van Merode F. (2014). Sustainability of improvements in access to outpatient specialist care in The Netherlands. *Journal of Health Services Research and Policy*.

[38] Mitchell V. (2008). Same-day booking: success in a Canadian family practice. *Canadian Family Physician*.

[39] Jennings M. E. (2014). Promoting patient satisfaction by utilizing a new scheduling concept: open access. *Entrepreneurship And Innovation Management*.

[40] Dixon S., Sampson F. C., O'Cathain A., Pickin M. (2006). Advanced access: more than just GP waiting times?. *Family Practice*.

[41] Knight A. W. (2009). Learning from four years of collaborative access work in Australia. *Quality in Primary Care*.

[42] Forjuoh S. N., Averitt W. M., Cauthen D. B., Couchman G. R., Symm B., Mitchell M. (2001). Open-access appointment scheduling in family practice: comparison of a demand prediction grid with actual appointments. *Journal of the American Board of Family Practice*.

[43] (2012). *Advanced access and efficiency workbook for primary care*.

[44] Steinbauer J. R., Korell K., Erdin J., Spann S. J. (2006). Implementing open-access scheduling in an academic family practice. *Family Practice Managment*.

[45] Rose K. D., Ross J. S., Horwitz L. I. (2011). Advanced access scheduling outcomes: a systematic review. *Archives of Internal Medicine*.

[46] Powell B. J., McMillen J. C., Proctor E. K. (2012). A compilation of strategies for implementing clinical innovations in health and mental health. *Medical Care Research and Review*.

[47] Pinto M. B., Parente D., Barber J. C. (2002). Selling open access health care delivery to patients and administrators: what's the hook?. *Health Marketing Quarterly*.

[48] (2016). *Practice Support program-Office Practice Redesign in Primary Health Care: Advanced Access and Office Efficiency Workbook*.

